# Innovative Biocompatible Blend Scaffold of Poly(hydroxybutyrate-co-hydroxyvalerate) and Poly(ε-caprolactone) for Bone Tissue Engineering: In Vitro and In Vivo Evaluation

**DOI:** 10.3390/polym16213054

**Published:** 2024-10-30

**Authors:** Amália Baptista-Perianes, Marcia Mayumi Omi Simbara, Sônia Maria Malmonge, Marcelo Rodrigues da Cunha, Daniela Vieira Buchaim, Maria Angelica Miglino, Elias Naim Kassis, Rogerio Leone Buchaim, Arnaldo Rodrigues Santos

**Affiliations:** 1 Centro de Ciências Naturais e Humanas (CCNH), Universidade Federal do ABC (UFABC), São Bernardo do Campo 09606-070, Brazil; amaliabaptistamp@gmail.com; 2 Centro de Engenharia, Modelagem e Ciências Sociais Aplicadas, Universidade Federal do ABC (UFABC), São Bernardo do Campo 09606-070, Brazil; marcia.simbara@ufu.br (M.M.O.S.); sonia.malmonge@ufabc.edu.br (S.M.M.); 3 Postgraduate Program in Health Sciences, Faculty of Medicine of Jundiaí (FMJ), Jundiaí 13202-550, Brazil; marcelocunha@g.fmj.br; 4 Postgraduate Program in Structural and Functional Interactions in Rehabilitation, Postgraduate Department, University of Marilia (UNIMAR), Marilia 17525-902, Brazil; danibuchaim@alumni.usp.br (D.V.B.); miglino@usp.br (M.A.M.); 5 Graduate Program in Anatomy of Domestic and Wild Animals, Faculty of Veterinary Medicine and Animal Science, University of São Paulo (FMVZ/USP), São Paulo 05508-270, Brazil; rogerio@fob.usp.br; 6 Medical School, University Center of Adamantina (UNIFAI), Adamantina 17800-000, Brazil; 7 Postgraduate Program in Animal Health, Production and Environment, University of Marilia (UNIMAR), Marilia 17525-902, Brazil; 8 University Center of the North of São Paulo (UNORTE), São José Do Rio Preto 15020-040, Brazil; eliascasa@terra.com.br; 9 Department of Biological Sciences, Bauru School of Dentistry (FOB/USP), University of São Paulo, Bauru 17012-901, Brazil

**Keywords:** poly(hydroxybutyrate-co-hydroxyvalerate), poly(ε-caprolactone), tissue engineering, bioresorbable polymers, mesenchymal stem cells, polymers

## Abstract

This study evaluated the biocompatibility of dense and porous forms of Poly(hydroxybutyrate-co-hydroxyvalerate) (PHBV), Poly(ε-caprolactone) (PCL), and their 75/25 blend for bone tissue engineering applications. The biomaterials were characterized morphologically using scanning electron microscopy (SEM) and Fourier transform infrared spectroscopy (FTIR), and the thickness and porosity of the scaffolds were determined. Functional assessments of mesenchymal stem cells (MSCs) included the MTT assay, alkaline phosphatase (ALP) production, and morphological and cytochemical analyses. Moreover, these polymers were implanted into rats to evaluate their in vivo performance. The morphology and FTIR spectra of the scaffolds were consistent with the expected results. Porous polymers were thicker than dense polymers, and porosity was higher than 92% in all samples. The cells exhibited good viability, activity, and growth on the scaffolds. A higher number of cells was observed on dense polymers, likely due to their smaller surface area. ALP production occurred in all samples, but enzyme activity was more intense in PCL samples. The scaffolds did not interfere with the osteogenic capacity of MSCs, and mineralized nodules were present in all samples. Histological analysis revealed new bone formation in all samples, although pure PHBV exhibited lower results compared to the other blends. In vivo results indicated that dense PCL and the dense 75/25 blend were the best materials tested, with PCL tending to improve the performance of PHBV in vivo.

## 1. Introduction

Autologous bone grafts are the gold standard for bone reconstruction surgery. However, the limited availability of this type of bone and the donor site morbidity have stimulated interest in developing new methods for effective bone repair [1]. In this context, tissue engineering has developed biomaterials that promote the rapid proliferation of bone cells, thus becoming an interesting alternative for regenerative medicine [2,3,4].

Among the materials developed for tissue engineering, temporary polymeric substances have been extensively studied. Bioresorbable polymers, in particular, are of great biomedical interest [5,6,7]. These materials are eliminated from the body through physiological processes such as metabolism or excretion [6]. Therefore, a second surgery for the removal of the material is unnecessary. The degradation of the scaffold increases the rates of cell proliferation and migration for subsequent replacement of newly formed tissue [7].

Many polymers have been tested for bone tissue engineering, with poly(hydroxybutyrate-co-hydroxyvalerate) (PHBV) being particularly noteworthy. This natural thermoplastic, produced by bacteria, has been experimentally used as an implant to repair small bone fractures and spinal cord injuries, showing good biocompatibility [8,9]. Classical experiments by Köse et al. [10] showed that osteoblasts cultured on PHBV matrices maintain their bone differentiation patterns. The results were highly satisfactory, with cells proliferating on the scaffold and producing large amounts of alkaline phosphatase (ALP) and osteocalcin. Moreover, osteoblasts were found to grow inside the matrices and promote their mineralization. Other studies evaluating the behavior of mesenchymal stem cells (MSCs) on fibrous PHBV scaffolds reported positive results in terms of cell viability. Furthermore, MSCs responded differently to substrate characteristics, such as PHBV fiber orientation [11]. Several assays for bone reconstruction using PHBV-based substrates indicated MSC growth in vitro and regenerative capacity in vivo [12].

Another widely used polymer is poly(ε-caprolactone) (PCL). This polyester is degraded by hydrolysis at physiological pH, is easily processed, and exhibits good compatibility with human tissues and good thermal stability [13]. Moreover, PCL is approved by the FDA (Food and Drug Administration, USA), making it an interesting material for tissue engineering applications [2,14]. MSCs show a good growth pattern on PCL. In a recent study, MSCs adhered to and proliferated easily on both pure and functionalized PCL scaffolds [15]. Expression of bone differentiation factors such as ALP, osteopontin, and calcium minerals was observed in all substrates [15].

The development of polymer blends aims to enhance their functions and characteristics, optimizing their use in tissue engineering. PHBV-based scaffolds have good tenacity, an important characteristic for bone tissue engineering, and can be combined with the plasticizing and biocompatible properties of PCL. In this respect, the properties of PHBV and PCL may complement each other [13], making PHBV-PCL blends a promising alternative. Some of the early reports on PHBV-PCL blends came from our research group. Over time, we have aimed to develop a porous scaffold for use in bone tissue engineering. Although recent studies have explored this blend, they have focused on different purposes and compositions. The physical characterization of these blends was previously reported [13]. Recent findings have shown that it is possible to obtain membranes of these blends with good porosity, no cytotoxicity, and the ability to actively support fibroblast cell cultures [16]. Moreover, dense PHBV and PCL membranes do not interfere with the osteogenic differentiation pattern of MSCs [17]. Finally, the 75/25 blends have shown the best results in previous studies [16]. In this study, we evaluated dense and porous forms of pure PHBV, PCL, and their 75/25 blend for bone tissue engineering using in vitro (MSCs) and in vivo (rat implants) assays.

## 2. Materials and Methods

### 2.1. Preparation of Dense and Porous PHBV, PCL, and PHBV/PCL (75/25) Blend Scaffolds

**Figure 1:**
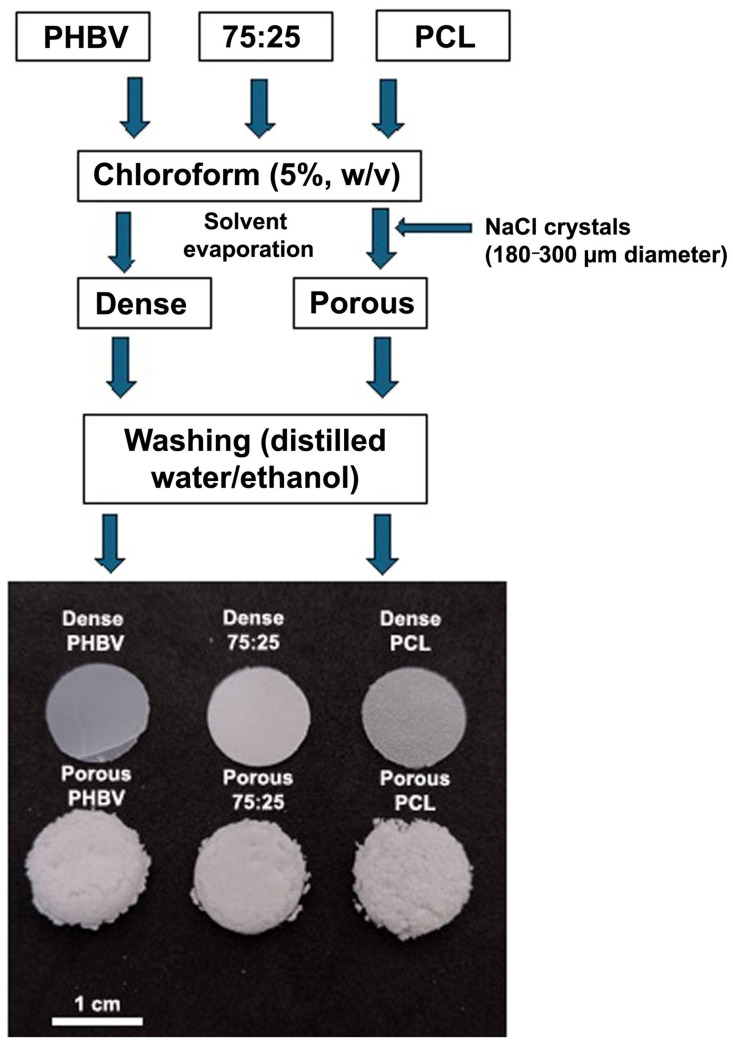
Schematic diagram of scaffold preparation.

In this study, we used PHBV (12% wt, Sigma-Aldrich^®^, 403121-100G, St. Louis, MO, USA), PCL (Sigma-Aldrich^®^, 440744-250G, Mn 70000–90000, St. Louis, MO, USA), chloroform (Synth^®^, Diadema, Brazil), NaCl, and ethanol (Vetec^®^, São Paulo, Brazil). Dense membranes of PHBV, PCL, and the 75/25 blend were prepared using the solvent casting technique [16]. For each membrane, a polymer solution in chloroform (5% *w*/*v*) was cast into Petri dishes and allowed to evaporate in a fume hood at room temperature. Porous scaffolds with the same composition were prepared by adding NaCl crystals (180–300 µm diameter) into the polymer solution (50% *w*/*v*, 1 g NaCl per mL of polymer solution), which was also cast into Petri dishes. After solvent evaporation, the scaffolds were washed with distilled water for 48 h to remove the salt. Finally, the scaffolds were washed with ethanol for 24 h and left to air dry (Figure 1). The tests conducted in this study followed the ASTM F813-83 guidelines [18] for the evaluation of biomaterials according to international standards [19]. Before cell inoculation, the polymers were disinfected in 70% ethanol (overnight at room temperature), immersed in medium 199 (Lonza) without fetal bovine serum (FBS), and incubated at 37 °C for 24 h.

### 2.2. Characterization of the Scaffolds

The thickness of the membranes of the pure polymers and the blend was measured using a caliper and characterized by scanning electron microscopy (SEM) and Fourier transform infrared spectroscopy (FTIR). For SEM, the samples were examined using a compact scanning electron microscope (JSM-6010LA, JEOL^®^, Peabody, MA, USA). For FTIR, the samples were analyzed with a spectrometer (Thermo Nicolet^®^ 6700, Madison, WI, USA) at a resolution of 2 cm^−1^. The pore diameter was determined using ImageJ^®^ software (version 1.48, Bethesda, MD, USA). Porosity was calculated using the following equation: p (%) = (p_p_ − p_s_)/p_s_ × 100 [20], where ρ_p_ = pore density of the non-porous polymer and p_s_ = pore density of the porous polymer.

### 2.3. Culture of Rat Mesenchymal Stem Cells (MSCs)

We used Gibco^®^ Rat (SD) Mesenchymal Stem Cells (S1601-100). These cells express a flow cytometry cell-surface protein profile positive for CD29, CD44, CD90, and C106 (>70%) and negative for CD11b, CD34, and CD45 (<5%). The cells were cultured in Dulbecco’s Modified Eagle’s Medium (DMEM) with low glucose and GlutaMAX^®^-I, supplemented with 10% MSC-Qualified FBS (Gibco^®^, Carlsbad, CA, USA) to promote optimal growth and maintain the undifferentiated state of MSCs. Cultures were maintained in a controlled environment at 37 °C with 5% CO_2_ and 95% humidity. For osteogenic differentiation, we used StemPro^®^ Osteocyte/Chondrocyte Differentiation Basal Medium (Gibco^®^, Carlsbad, CA, USA) according to the manufacturer’s recommendations.

### 2.4. MTT Assays

Two MTT (3-(4,5-dimethylthiazol-2-yl)-2,5-diphenyl tetrazolium bromide) assays were conducted to evaluate the viability of Vero cells cultured on dense and porous materials. Samples of each polymer (*n* = 5) were placed in 96-well plates (Corning, Corning, NY, USA). Next, 200 µL of a cell suspension (2 × 10^5^ cells/well) was inoculated into the materials. The plates were incubated at 37 °C with 5% CO_2_ and 95% humidity for 24 h. After this period, 10 µL of MTT was added to each well, and the plates were incubated under the same conditions for an additional 4 h in the dark. Then, 100 µL of 10% sodium dodecyl sulfate (SDS) was added to each well to lyse the cell membranes. Absorbance was measured after 12 h using a DNM 9602 Microplate Reader^®^ (Beijing Perlong Technology, Beijing, China) at a wavelength of 570 nm. Wells containing only medium 199 without serum and the same reagents served as blanks. Wells containing materials but no cells served as controls for the reaction, whereas wells containing cells but no materials served as positive controls. Wells with cells treated with 70% alcohol to reduce viability were used as negative controls. Statistical analysis was conducted using one-way ANOVA followed by Tukey’s post-test, with a significance level of 5%.

### 2.5. Morphological Analysis

For the direct contact test, the polymers were cut into appropriate sizes and placed in a 24-well plate. MSCs were inoculated at a concentration of 2 × 10^5^ cells/well (1.5 mL) onto the pure polymers and PHBV/PCL blends (dense and porous samples). The plate itself served as the control. The cells were incubated in DMEM with low glucose and GlutaMAX-I (Gibco^®^, Carlsbad, CA, USA), supplemented with 10% FBS, at 37 °C with 5% CO_2_. After 5 days, the medium was removed from the wells, and the samples were fixed in 10% formalin (in 0.1 M phosphate-buffered saline—PBS, pH 7.2) within the culture plate and washed twice with distilled water. The cells were then stained with crystal violet.

### 2.6. Analysis of Osteogenic Differentiation: Alkaline Phosphatase (ALP) and Alizarin Red Staining (ARS)

MSCs cultured under osteogenic differentiation conditions were analyzed for ALP production and ARS. ALP production was assessed using a specific kit (Sigma Fast p-nitrophenyl phosphatase) following the manufacturer’s recommendations. Briefly, 50 µL of differentiation medium from the 24-well plate was transferred to a 96-well plate and incubated for 2 h at 37 °C. Next, 200 µL of ALP substrate solution was added, and the plate was incubated in the dark for 30 min at room temperature. Absorbance was measured at 405 nm. The analysis was performed using media conditioned by cells that grew in contact with the tested materials (including controls) after 7, 14, and 21 days of incubation.

For ARS, after 21 days of incubation, the cells were fixed in 4% paraformaldehyde for 10 min, washed with distilled water, and stained for 2 min. The cells were washed again with distilled water and differentiated in 95% ethanol and 100% hydrochloric acid for 15 s. The cells were kept in distilled water while images were obtained with a Leica inverted microscope (Leica TCS-SPE^®^, Mannheim, Germany). ARS activity was evaluated using the semiquantitative colorimetric method described by Gregory et al. [21]. After discarding the water, the plate was kept at room temperature until fully dry. Next, 10% acetic acid was added, and the plate was shaken for 30 min. The contents of each well were transferred to Eppendorf tubes, heated to 85 °C for 10 min, and then cooled on ice for 5 min. Next, the tubes were centrifuged at 16,000× *g* for 20 min, and the supernatant was transferred to a new tube containing 10% ammonium hydroxide.

### 2.7. Cytochemical Analysis of MSCs Cultured Under Osteogenic Differentiation Conditions

After 21 days of culture, MSCs cultured under osteogenic differentiation conditions were stained with toluidine blue (TB) at pH 4.0 to identify DNA, RNA, and glycosaminoglycans (GAGs) and with xylidine ponceau (XP) at pH 2.5 to identify total proteins [22,23]. The samples were analyzed using a light microscope (Nikon^®^, model 80i, Tokyo, Japan) with a 20× objective. Images were captured with a Nikon Digital Sight camera (DS-Ri1, Nikon^®^, Tokyo, Japan) coupled to the microscope using the NIS-Elements software v. 5.21.00 (Nikon Instruments^®^, Tokyo, Japan). Because of the irregularities of the substrates, 4 to 12 images of the same field were obtained for each material, photographed under a 4× objective at intervals of 0.5 µm. For the 40× objective, images were captured at intervals of 0.2 µm. All images were merged using the Combine ZP software v. 1.0, and the final images were analyzed using the Infinity Analyze software v. 6 (Teledyne Lumenera^®^, Ottawa, ON, Canada).

### 2.8. Scanning Electron Microscopy (SEM) of Cellularized Scaffolds

The materials were placed in a 24-well culture plate, and a cell suspension was inoculated at a concentration of 2 × 10^5^ cells/well (1.5 mL). The plate was then incubated for 21 days at 37 °C. After incubation, the samples were fixed for 2 h at room temperature in a 3% glutaraldehyde solution (Sigma-Aldrich^®^, St. Louis, MO, USA) dissolved in 0.1 M cacodylate buffer (pH 7.4). The materials were subsequently washed with PBS, post-fixed with 1% osmium tetroxide (Sigma-Aldrich^®^, St. Louis, MO, USA) in a refrigerator for 15 min, washed with distilled water, and dehydrated in increasing concentrations of ethanol (50%, 70%, 95%, and 100%). After that, the samples were dried using a critical point dryer (Leica EM CPD300^®^, Wetzlar, Germany), coated with gold (Sputtering Leica ACE 200^®^, Leica Microsystems, Wetzlar, Germany), and observed under a scanning electron microscope (FEI Quanta 250^®^, Hillsboro, OR, USA).

### 2.9. Animals and Surgical Procedure

Forty-two male Wistar rats (*Rattus norvegicus*), 4 months old, with an average weight of approximately 365 g, were maintained at the bioterium of the School of Medicine of Jundiaí (FMJ/Jundiaí, Brazil). This experimental protocol was approved by the Ethics Committee on Animal Experimentation of FMJ (Protocol 49/2018). The rats were housed individually in appropriate cages, with a temperature of approximately 23 °C and a light/dark cycle controlled by a timer device set for 12 h each. All specimens received food (Nuvilab^®^, Curitiba, Brazil) and water ad libitum. We followed the existing ARRIVE guidelines for in vivo experimentation to monitor all stages of the experiment.

The animals were anesthetized with an intramuscular injection of a 1:1 mixture of ketamine hydrochloride (Francotar^®^, Virbac, São Paulo, Brazil) and xylazine hydrochloride (Virbaxyl^®^, Virbac, São Paulo, Brazil) at a dose of 0.010 mL per 10 g of body weight. A trichotomy was performed in the proximal tibia region, and the skin was cut longitudinally and separated laterally. A 3.0 mm surgical bur coupled to a handheld drill (Beltec LB-100^®^, Araraquara, Brazil) was used to create a bone defect in the left proximal tibia. The defect was then filled with a 3.0 mm scaffold made of different biomaterials or left empty in the control group. The animals were randomly assigned to the following groups (*n* = 6 each): G1—control, untreated bone defect; G2—bone defect implanted with dense PHBV; G3—bone defect implanted with porous PHBV; G4—bone defect implanted with dense 75/25 blend; G5—bone defect implanted with porous 75/25 blend; G6—bone defect implanted with dense PCL; G7—bone defect implanted with porous PCL. The skin was closed using 4-0 silk sutures.

### 2.10. Histomorphometric and Statistical Analysis

The rats were euthanized 6 weeks after surgery using an excessive (triple) dose of the anesthetics described for the experimental surgery. The operated tibias were then removed and subjected to routine histological processing (decalcification and embedding in paraffin blocks for sectioning at 5 μm thickness). Histological sections of the entire wound area were stained with Masson’s trichrome to characterize and differentiate bone neoformation. The formation of new bone was quantified using Motic software (Motic Images Plus 2.0ML software^®^, Motic China Group, Xiamen, China). In each image, the total area of the osteotomy and the area of newly formed bone were delineated, and bone volume was calculated as a percentage. Values were compared between groups using ANOVA followed by Tukey’s post-test, with a significance level set at *p* < 0.05.

## 3. Results

### 3.1. Scaffolds

**Figure 2:**
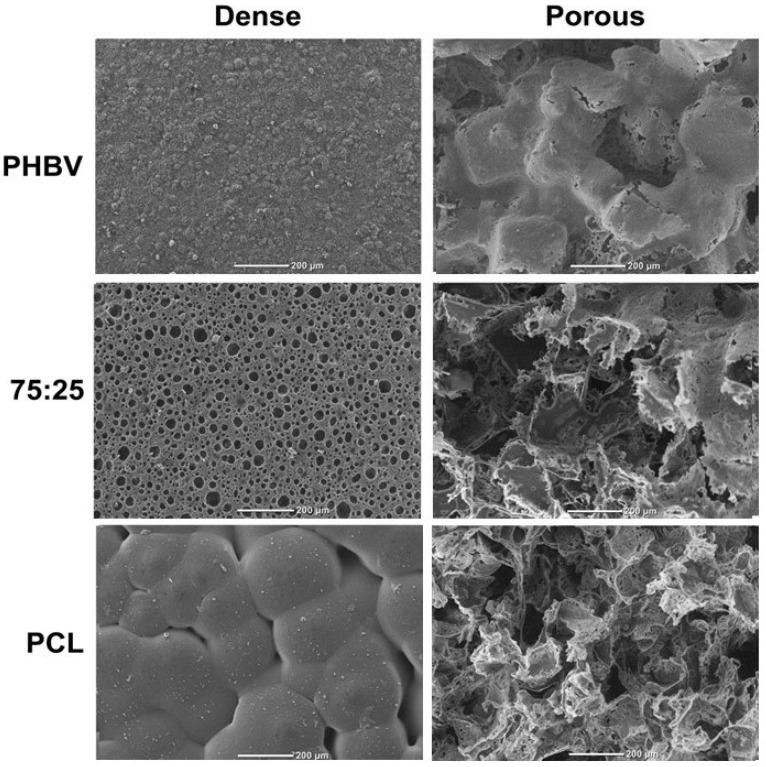
Scanning electron microscopy images of the upper surface of the studied materials [PHBV: poly(hydroxybutyrate-co-hydroxyvalerate; PCL: poly(ε-caprolactone); the 75/25 blend]. Scale bar = 200 µm.

Figure 2 illustrates the morphological characteristics of the dense and porous scaffolds as observed by SEM. Both dense and porous membranes presented irregular surfaces, with the porous membranes presenting irregularities in the shape of the NaCl crystals. For the PHBV/PCL (75/25) blend, the surface of the dense membranes contained small, homogeneously distributed pores, whereas the porous membranes exhibited irregular pores that extended to the surface. PCL scaffolds showed a granule-like structure on the surface of the dense membranes and a porous structure on the surface of the porous membranes. Table 1 summarizes the apparent porosity, pore diameter, and thickness of the scaffolds. Overall, the samples exhibited high porosity (>92%), with pore diameters ranging from 240 to 280 μm. The porous scaffolds were thicker than the dense ones.

### 3.2. FTIR Scaffolds

**Figure 3:**
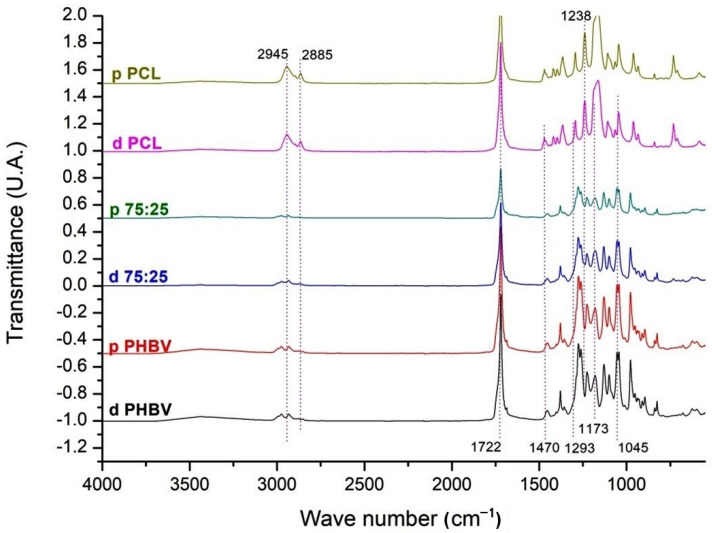
FTIR spectra of dense and porous PHBV, 75/25 blend, and PCL in the range of 4000 to 500 cm^−1^. d PHBV = dense PHBV (G2); p PHBV = porous PHBV (G3); d 75:25 = dense 75/25 blend (G4); p 75:25 = porous 75/25 blend (G5); d PCL = dense PCL (G6); p PCL = porous PCL (G7).

Figure 3 shows the FTIR spectra of PHBV, PCL, and the PHBV/PCL (75/25) blend. A small stretching vibration is observed around 668 cm^−1^, corresponding to the vibrational points of the carboxyl group (CH) bond present in both polymer chains. Another band appears at approximately 2885 cm^−1^, associated with the elongation of the carboxyl bond. In the region of 2865–2945 cm^−1^, bands related to both symmetrical and asymmetrical CH_2_ bonds are identified. The largest band is found at a wavelength of 1722 cm^−1^, corresponding to the carbonyl bond present in both polymers. Within the 1045–1470 cm^−1^ range, bands characteristic of carbonyl (C–O), carbon-carbon (C–C), and ether (C–O–C) bonds are observed at 1293, 1173, and 1238 cm^−1^, respectively. The PCL spectra better illustrate these data.

### 3.3. MTT Assay

**Figure 4:**
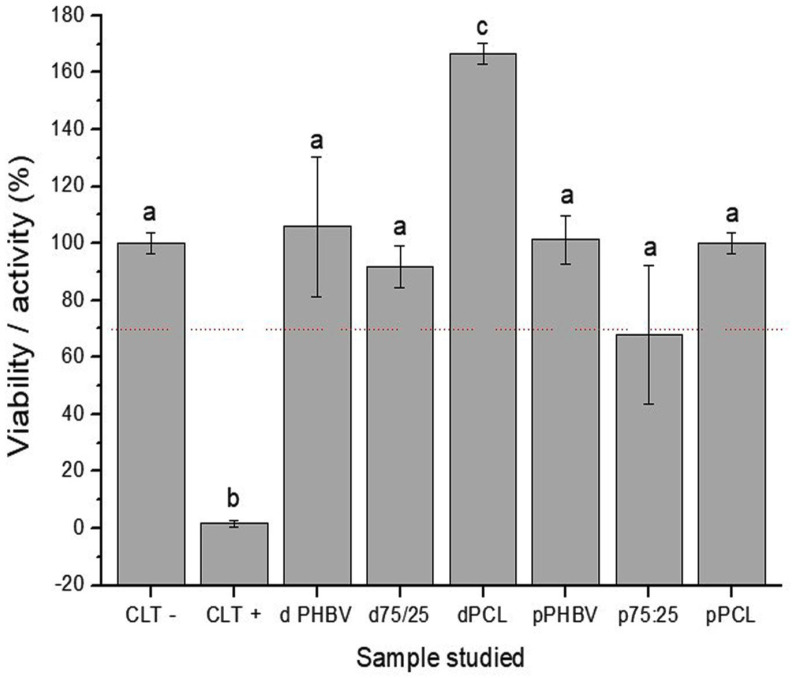
MTT assay results of mesenchymal stem cells after 24 h of incubation. Different lowercase letters indicate significant differences between groups (*p* < 0.05). CLT − = Negative Control; CLT + = Positive Control; d PHBV = dense PHBV (G2); p PHBV = porous PHBV (G3); d 75:25 = dense 75/25 blend (G4); p 75:25 = porous 75/25 blend (G5); d PCL = dense PCL (G6); p PCL = porous PCL (G7). The dashed line indicates a 30% reduction in viability of the negative control.

When cultured with MSCs, all substrates performed better than the negative control, except for the porous 75/25 blend. Statistically, the tested polymers were similar to the positive control, except for the dense PCL membrane, which outperformed the other substrates (Figure 4).

### 3.4. Morphological Analysis of MSCs

**Figure 5:**
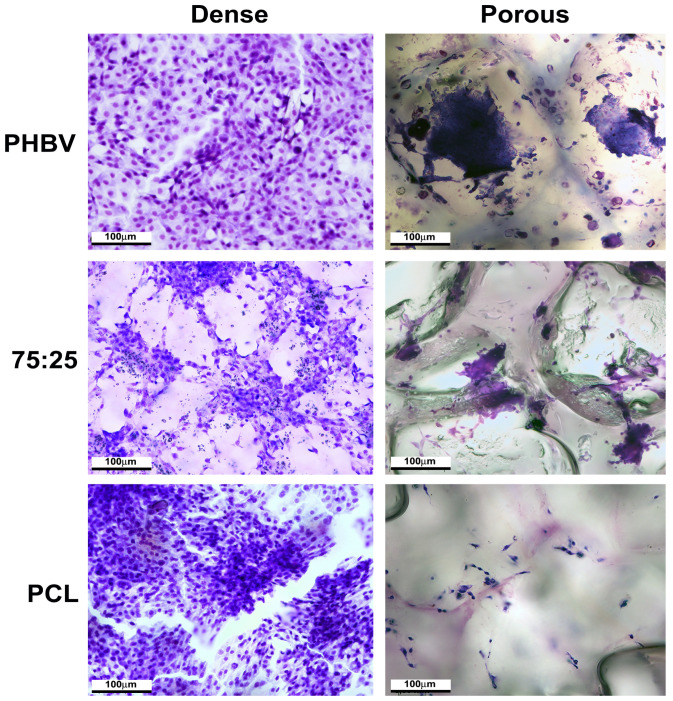
Morphological analysis of mesenchymal stem cells cultured on dense and porous polymers for 5 days. Stain: crystal violet. Scale bar = 100 μm.

Cells cultured on dense materials exhibited a confluent to semi-confluent distribution pattern. We observed cells with irregular morphology, nuclei with decondensed chromatin, and partially visible nucleoli. The number of MSCs was lower on porous than on dense samples. Elongated cells were the most prevalent cell type observed in the samples (Figure 5).

### 3.5. Analysis of Osteogenic Differentiation by Alkaline Phosphatase Measurements

**Figure 6:**
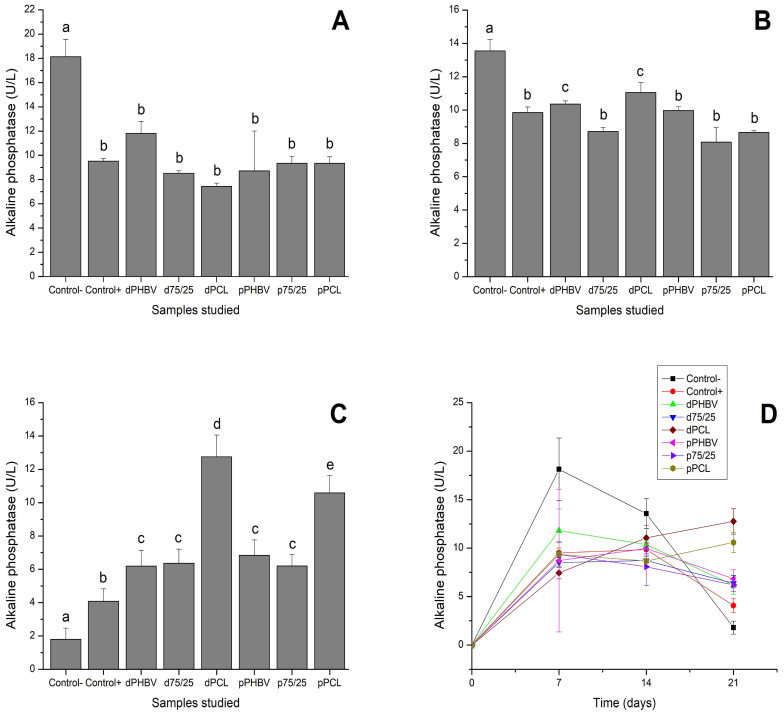
Production of alkaline phosphatase during osteogenic differentiation. (**A**) 7 days; (**B**) 14 days; (**C**) 21 days; (**D**) over time. Different lowercase letters indicate statistically significant differences (one-way ANOVA and Tukey’s post-test, *p* < 0.05). CLT − = Negative Control; CLT + = Positive Control; d PHBV = dense PHBV (G2); p PHBV = porous PHBV (G3); d 75:25 = dense 75/25 blend (G4); p 75:25 = porous 75/25 blend (G5); d PCL = dense PCL (G6); p PCL = porous PCL (G7).

At 7 days of differentiation, ALP peaked in the negative control (common medium), occurring earlier and at a higher level than observed in the samples. In the samples, ALP activity levels were similar to those in the positive control (induction medium) (Figure 6A). By 14 days (Figure 6B), ALP production appeared to decrease in the negative control but remained stable in the positive control and in the samples in which the cells grew in contact with the polymers. An exception was observed in dense PCL, which showed increased ALP activity compared to day 7. At 21 days, a marked decline in ALP activity was detected in the positive and negative controls. Although most polymers also exhibited reduced ALP activity compared to the 14-day time point, their levels remained higher than those of the controls. Notably, both dense and porous PCL maintained stable ALP production, with higher activity compared to earlier time points (Figure 6C). Figure 6D illustrates the temporal expression of ALP.

### 3.6. Analysis of Osteogenic Differentiation by Alizarin Red Staining

**Figure 7:**
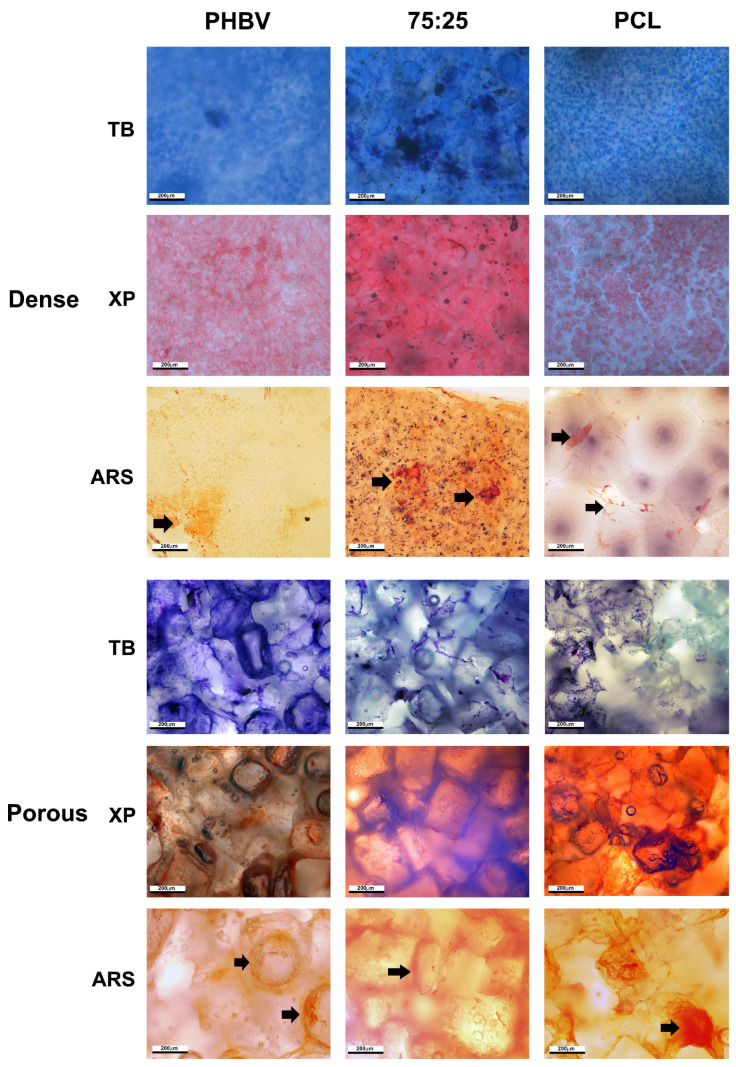
Cytochemical and osteogenic differentiation of mesenchymal stem cells (MSCs) cultured on dense and porous polymers for 21 days, as assessed by alizarin red staining (ARS). Black arrows indicate ARS marking. TB = Toluidin Blue at pH 4.0; XP = Xylidine Ponceau at pH 2.5; ARS = Alizarin Red Staining. Scale bar = 200 μm.

Mineralized nodules were observed in each image of all samples, except for the negative control (without osteogenic differentiation medium). Although the number of nodules was small, this finding confirms the differentiation capacity of the cells on the polymers (Figure 7).

### 3.7. Cytochemical Analysis of MSCs Under Osteogenic Differentiation Conditions

Cytochemical analysis was conducted after 21 days of culture, and the results are presented in Figure 6. Staining with TB at pH 4.0 revealed metachromatic cytoplasm and orthochromatic nuclei in cells grown on porous samples. Cells on dense materials exhibited slightly metachromatic cytoplasm but maintained orthochromatic nuclei. Staining with XP at pH 2.5 revealed highly acidophilic cells on the materials, with a more intense staining pattern observed in dense samples because of the higher cell density. No cytochemical variations were noted among the different polymers (Figure 7).

### 3.8. Scanning Electron Microscopy (SEM) of Cellularized Scaffolds

**Figure 8:**
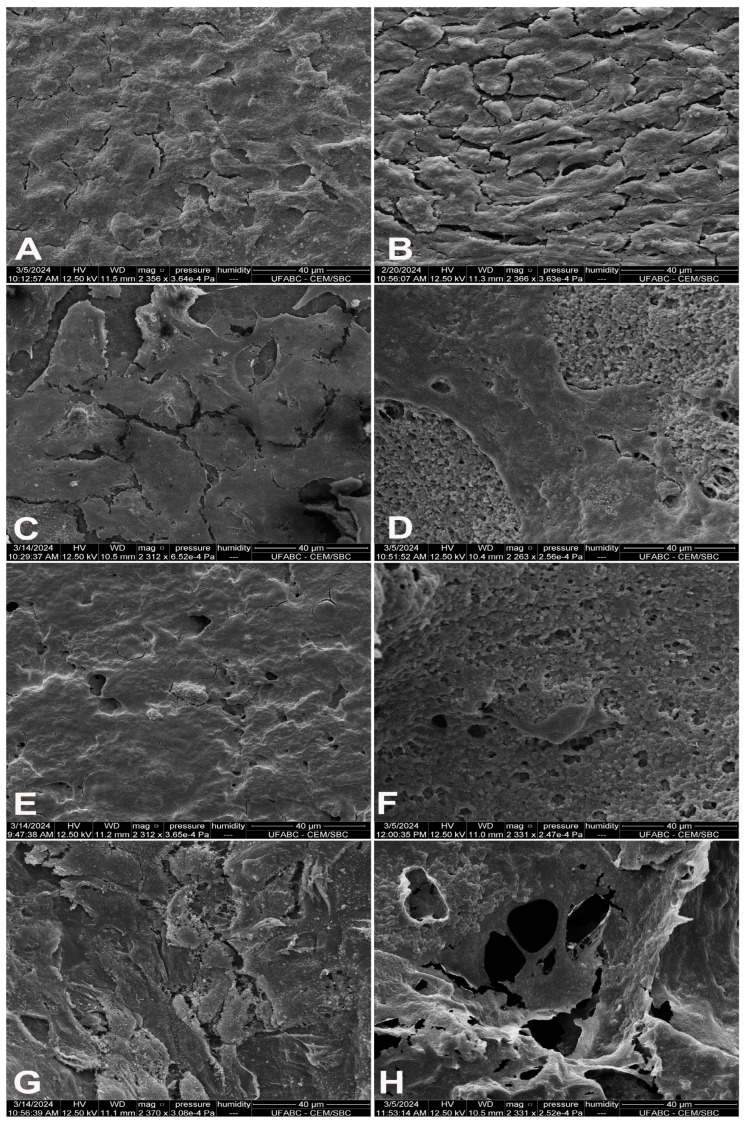
Scanning electron microscopy of mesenchymal stem cells cultured on dense and porous polymers for 21 days. (**A**) Culture plate without mineralization medium; (**B**) culture plate with mineralization medium; (**C**) dense PHBV; (**D**) porous PHBV; (**E**) dense 75/25 blend; (**F**) porous 75/25 blend; (**G**) dense PCL; (**H**) porous PCL. Scale bar = 40 μm.

In the monolayer regions, MSCs showed irregular morphology and slightly flattened cells in culture dishes (Figure 8A,B). No signs of mineralization were seen in these regions. Locating the cells in the porous samples was difficult; cells were identifiable only in regions of lower confluence. In the dense PHBV, the cells exhibited less elongation (Figure 8C). In the porous PHBV, a flat layer of cells adhered well to the material (Figure 8D). The dense 75:25 blend exhibited regions of semi-confluence and confluence, resulting in a homogeneous monolayer that was difficult to characterize. The characteristics of the material were visible in areas lacking cellular contact (Figure 8E). In the porous blends, the characteristics of the cells intermixed with the material surface, preventing a clear analysis (Figure 8F). Dense PCL exhibited either a semi-confluent or confluent layer, depending on the region (Figure 8G). Finally, in porous PCL, the cells around the pores of the material were clearly distinguishable (Figure 8H).

### 3.9. Histological and Morphometric Analysis

**Figure 9:**
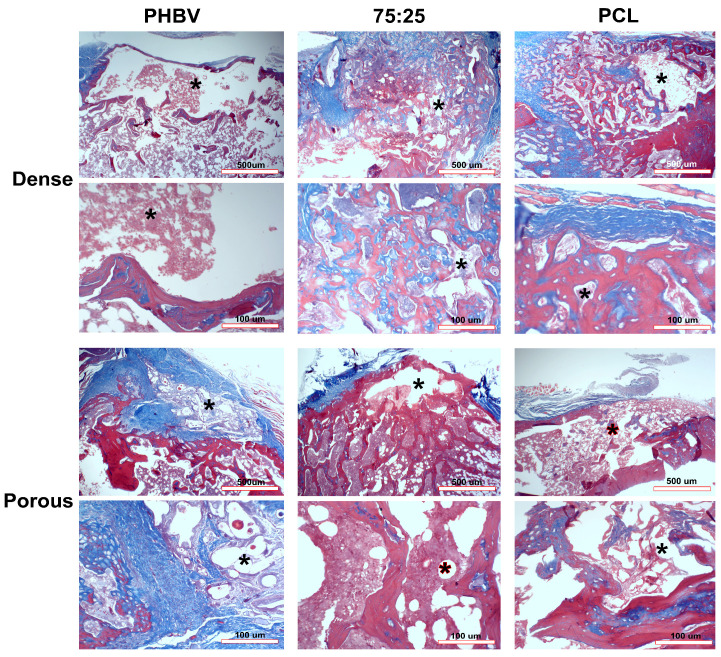
Histological analysis of the experimental groups stained with Masson’s trichrome. Asterisks indicate the implant site. Scale bars = 100 μm and 500 μm.

No tissue degeneration or signs of a local apoptotic process were observed at the implant site in any of the samples. In the control group (G1), minimal bone neoformation occurred solely at the defect borders, with the central area filled exclusively with dense connective tissue. In the dense PHBV group (G2), trabecular bone neoformation was observed at the defect borders, along with the presence of mature bone. Implant remnants were evident around the newly formed bone (Figure 9). In the porous PHBV group (G3), newly formed mature trabecular bone was found only at the defect borders, with material remnants covered by fibrous connective tissue (Figure 9). In both cases, no osseointegration between the polymer and bone was observed. 

In the dense 75/25 blend group (G4), immature bone formed mainly at the defect borders, but some trabecular bone also formed in the center of the defect. Fragments of the membrane were located centrally, bordered by newly formed bone and connective tissue. Areas of mature bony trabeculae were also observed in the center of the defect (Figure 9). In the porous 75/25 blend group (G5), mature trabecular bone formed around the implant, whereas remnants of the material were present in the more central area of the defect (Figure 9). In the dense PCL group (G6), there was a predominance of immature and irregular newly formed bone around the implant, with more mature bone observed near the defect borders. Membrane remnants were observed in the center of the defect, interspersed with newly formed bone and fibrous connective tissue (Figure 9).

In the porous PCL group (G7), both mature and immature bone formed from the margins of the defect. Remnants of the material were also observed in the center, interspersed with newly formed bone and connective tissue. The trabeculae of the newly formed bone showed characteristics of mature bone (Figure 9).

**Figure 10:**
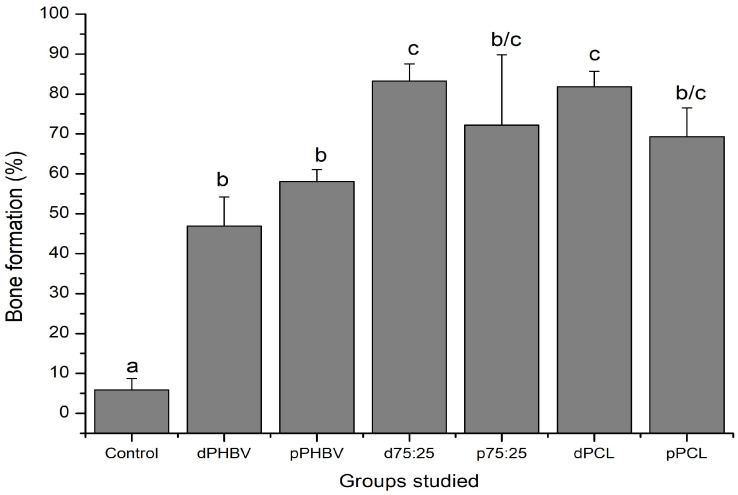
Morphometric analysis of bone formation (%) across experimental groups. Different lowercase letters indicate significant differences among groups (one-way ANOVA and Tukey’s post-test, *p* < 0.05). Control (G1); dPHBV = dense PHBV (G2); pPHBV = porous PHBV (G3); d75:25 = dense 75/25 blend (G4); p75:25 = porous 75/25 blend (G5); dPCL = dense PCL (G6); pPCL = porous PCL (G7).

Regarding morphometry, the PHBV implants (dense, G2 and porous, G3) yielded the poorest results among the materials studied. The largest amount of newly formed bone was observed in the dense 75/25 blend (G4) and dense PCL (G6). Unfortunately, the results obtained for the porous blend (G5) and PCL (G7) were inconclusive because of the high standard deviations; however, they indicated bone formation of approximately 70% (Figure 10).

## 4. Discussion

Bone defects generally arise from the loss of bone mass due to trauma, pathological fractures, abnormal skeletal development, or tumor resection. Fractures accompanied by bone mass loss often exceed the regenerative capacity of the individual and require the use of grafts or implants. Tissue engineering has developed biomaterials that stimulate bone regeneration and have thus become an interesting alternative in regenerative medicine [2]. Various PHBV/PCL ratios have been previously tested [13], and physical characterizations identified the 50:50 and 75:25 blends as the most suitable for cytotoxicity studies. However, the 50:50 blend exhibited a low cellular response [16], and ultimately, the dense 75:25 blend might be suitable for MSC culture [17]. Here, we assessed the use of pure PCL and PHBV scaffolds, as well as their 75/25 blend, for MSC culture.

The morphological characteristics of the tested biomaterials are consistent with previously reported results for PHBV [24], PCL, and PHBV/PCL blends [13,16]. For the porous polymers, SEM images revealed an irregular surface with well-distributed pores of varying sizes and no pore-free areas—an important characteristic for cell culture [25]. Although the optimal pore size for bone growth remains debated (with recommended ranges between 100 and 500 µm [26,27]), the pore sizes observed here fall within the recommended range of 200–300 µm [28,29]. FTIR characterization confirmed that the constructed scaffolds are compatible with the polymers used [13]. Moreover, the physical characteristics of the blends tested here were consistent with those reported in previous studies [13,17]. Our results are also consistent with recent findings on PHBV/PCL blends of various proportions [30].

In the MTT assay, MSCs exhibited higher activity after 24 h of incubation across all polymers compared to the negative control. Notably, the porous 75/25 blend approached the toxicity threshold defined by ISO standards. However, cells were present on the samples after 5 and 15 days of culture, indicating that the porous 75/25 blend cannot be classified as toxic; instead, the cells exhibited lower or slower initial activity. In contrast, dense PCL exhibited higher activity and viability, a result not seen with fibroblast cells [16,17]. Nevertheless, this increased activity did not reflect in a higher cell number at later time points or in the amount of bone formed in the implants. Although good adhesion and viability of endothelial cells on PHBV/PCL fibrous blends have been previously demonstrated [31], those results are not directly comparable to ours, as the cited study included VEGF addition to the scaffold.

After 5 days of incubation, dense samples exhibited a confluent to semi-confluent cellular monolayer, whereas porous samples had fewer cells. This result was expected because porous materials, due to their irregularities, have a larger surface area than dense materials; consequently, there is more space for cell colonization over the same time frame. This finding was corroborated by both SEM and ARS assays, despite the extended cultivation period. The morphological patterns of the cells on the tested materials are compatible with previous results involving fibroblasts [16] or MSCs [17]. Studies on osteosarcoma cell adhesion to fibrous PHBV/PCL blends also reported higher cell density in areas with smaller pores, offering more surface area for cell growth. However, because these blends were enriched with mineral components, direct comparison is unfeasible [32]. The pattern of mineral deposition observed through ARS for MSCs growing around PHBV/PCL blends was consistent with the existing literature [33].

Functional assessment of MSCs was conducted 21 days after the induction of osteogenic differentiation through ALP measurement and cytochemical analysis. ALP plays an important role in osteogenic differentiation. Based on the results of ALP expression, the various polymers tested did not appear to interfere with the osteogenic differentiation process, except for the PCL samples, in which ALP production increased over the incubation period. The kinetics of ALP production in PHBV/PCL blends aligns with previous reports [32]. This enzyme is an important component of the bone matrix, catalyzing calcium nucleation in the organic matrix and serving as an indicator of osteoblast activity and differentiation [34]. Moreover, extracellular ALP activity is an important parameter for assessing osteogenic potential both in vivo and in vitro [35]. Our data support this hypothesis, as osteogenic differentiation was confirmed by the presence of mineralized nodules on the polymers, usually detected after 28 days of incubation. Previous studies have also confirmed osteogenic differentiation through mineral deposition at this time point using the same dense materials [17]. Our goal was to obtain this information at the last ALP measurement. Although it was early in the process, we observed mineralized nodules on the samples.

Cytochemical analysis using TB staining at pH 4.0 revealed strongly metachromatic cells. TB is a basic dye that forms electrostatic interactions with acidic radicals in tissues, resulting in a purplish-blue stain for acidic structures. It binds to PO_4_^−^, SO_4_^−^, and COO^−^ groups, which are present in nucleic acids (DNA and RNA) and GAGs. An advantage of TB staining is its ability to provide indirect information about cell activity; a highly basophilic cytoplasm typically indicates a high amount of cytoplasmic rRNA, which is associated with the rough endoplasmic reticulum [22,23]. Staining after the induction of differentiation revealed a similar pattern and distribution of cells on both dense and porous materials after 5 days of culture, although a higher number of cells were observed on the scaffolds. Despite osteogenic differentiation, cell proliferation on the materials did not appear to be reduced. Intense staining of MSCs on the dense polymers indicated a higher number of cells.

XP is an acid dye that, at pH 2.5, stains cationic radicals, specifically cytochemically detectable NH_3_^+^ radicals, present in total proteins [22,23]. The cytochemical analysis indicated high protein deposition on all polymers (both dense and porous) following the induction of differentiation. These results suggest that MSCs can grow on the polymers and exhibit high functional activity, indicating their potential for osteogenic differentiation even at an early stage. Collectively, the in vitro data indicate that PCL performed the best, showing the highest activity in the MTT assay and the greatest ALP production. Interestingly, PHBV/PCL scaffolds supported chondrogenic differentiation better than the other materials studied, as evidenced by soluble GAG analysis and microscopic observations of chondrogenic matrix deposition [36].

In the in vivo assays, we used Masson’s trichrome staining to qualitatively analyze bone morphology. This method differentiates original mature bone, which stains red, from immature newly formed bone, which stains blue. Moreover, this method identifies collagen fibers in the extracellular matrix of fibrous tissues, which also stain blue [37,38,39]. We observed that bone neoformation progressed from the margins of the defect toward the center. None of the samples exhibited tissue degeneration at the implant site or signs of local apoptosis. Some studies indicate that PHBV implants are less likely to elicit an inflammatory response and fibrous connective tissue formation compared to other materials. Increased bone mineral density has been reported for PHBV implants, stimulating bone tissue regeneration in bone defects in vivo [40]. PHBV was found to induce a mild immune response in vivo, with evidence of macrophage infiltration. The extent of this inflammation diminished within 4 weeks post-surgery. Bone formation occurred adjacent to the implant and appeared unaffected by PHBV [41]. Our findings are consistent with these studies, although they did not quantify the newly formed bone. We observed the lowest level of bone formation for PHBV, ranging from 45% to 57%.

Park et al. [42] implanted PCL scaffolds into alveolar bone defects along with β-tricalcium phosphate (β-TCP) powder. Their in vivo analysis showed that the PCL implants preserved bone conductivity around the defects, with no inflammatory infiltrates observed. Bone neoformation occurred adjacent to the scaffolds, accounting for approximately 25% of the newly formed bone. Our findings align with those of Park et al. [42], although we observed more intense bone formation. Another study investigated the bone-healing capacity of PCL/PLGA scaffolds in bone defects in rabbits. X-ray, micro-CT, biochemical, and histological analyses revealed that PCL/PLGA scaffolds promote bone neoformation by inducing repair, suggesting they may be a good option for fracture treatment [43]. Our data indicate that PCL, whether used alone or blended with PHBV, is more effective than when combined with PLGA.

In rabbit models, the nanocomposite PCL group exhibited a larger amount of newly formed lamellar bone compared to the other groups, suggesting that PCL is a promising material for bone implants [44,45,46]. The PCL scaffold also yielded satisfactory results for repairing bone defects around dental implants. The incorporation of β-TCP, MSCs, and platelet-rich plasma (PRP) optimized the bone regeneration process and improved implant stability [47,48,49,50].

Possible limitations of this study include the fact that it involved only small animals and the absence of mechanical resistance tests on the newly formed bone in the evaluated biomaterials. To develop a device suitable for human patients, it will be necessary to understand how these blends behave in biological models other than murine rodents. Moreover, the biomechanical properties of the newly formed bone must be thoroughly evaluated in future studies.

## 5. Conclusions

In this study, the physical characterization of the scaffolds was consistent with previous studies. The cells exhibited good viability and growth on the produced scaffolds. The growth was more pronounced in dense samples due to their smaller surface area. The scaffolds did not interfere with the osteogenic capacity of MSCs, and microscopic observations showed no tissue degeneration or signs of local apoptosis at the implant sites. New bone formation occurred in all samples, although pure PHBV showed lower results compared to the other blends. Our in vivo findings identified dense PCL and the dense 75/25 blend as the best materials. For the porous materials, our data were inconclusive. PCL tended to improve the performance of PHBV in vivo. The addition of this polymer to PHBV blends improved the in vivo results compared to the pure polymer. Future studies should focus on further testing these biomaterials, particularly in medium and large animal models, to translate these findings into clinical applications in regenerative dentistry and medicine.

## Figures and Tables

**Table 1:** Thickness, porosity, and pore diameter of PHBV, 75/25, and PCL scaffolds.

Samples		Thickness (μm)	Porosity (%)	Pore Diameter (μm)
PHBV	Dense	190 ± 48	-	-
	Porous	2500 ± 280	92.38 ± 0.20	280 ± 100
75:25	Dense	194 ± 41	-	-
	Porous	2157 ± 410	93.17 ± 0.34	245 ± 130
PCL	Dense	197 ± 6	-	-
	Porous	1997 ± 520	93.68 ± 0.87	242 ± 51

PHBV—Poly(hydroxybutyrate-co-hydroxyvalerate); PCL—Poly(ε-caprolactone).

## Data Availability

The original contributions presented in the study are included in the article, further inquiries can be directed to the corresponding author.

## References

[1] Bharadwaz A., Jayasuriya A.C. (2020). Recent trends in the application of widely used natural and synthetic polymer nanocomposites in bone tissue regeneration. Mater. Sci. Eng. C Mater. Biol. Appl..

[2] Siddiqui N., Kishori B., Rao S., Anjum M., Hemanth V., Das S., Jabbari E. (2021). Electropsun polycaprolactone fibres in bone tissue engineering: A review. Mol. Biotechnol..

[3] Zoghi S. (2024). Advancements in tissue engineering: A review of bioprinting techniques, scaffolds, and bioinks. Biomed. Eng. Comput. Biol..

[4] Li G., Gao F., Yang D., Lin L., Yu W., Tang J., Yang R., Jin M., Gu Y., Wang P. (2024). ECM-mimicking composite hydrogel for accelerated vascularized bone regeneration. Bioact. Mater..

[5] Santos A.R., Zavaglia C.A.C., Hashmi S. (2016). Tissue Engineering Concepts. Reference Module in Materials Science and Materials Engineering.

[6] Vert M., Doi Y., Hellwich K.H., Hess M., Hodge P., Kubisa P., Rinaudo M., Schué F. (2012). Terminology for biorelated polymers and applications (IUPAC Recommendations 2012). Pure Appl. Chem..

[7] Saska S., Pilatti L., Blay A., Shibli J.A. (2021). Bioresorbable polymers: Advanced materials and 4D printing for tissue engineering. Polymers.

[8] Li J., Zhang X., Udduttula A., Fan Z.S., Chen J.H., Sun A.R., Zhang P. (2021). Microbial-Derived Polyhydroxyalkanoate-Based Scaffolds for Bone Tissue Engineering: Biosynthesis, Properties, and Perspectives. Front. Bioeng. Biotechnol..

[9] Elmowafy E., Abdal-Hay A., Skouras A., Tiboni M., Casettari L., Guarino V. (2019). Polyhydroxyalkanoate (PHA): Applications in drug delivery and tissue engineering. Expert Rev. Med. Devices.

[10] Köse G.T., Korkusuz F., Korkusuz P., Purali N., Özkul A., Hasırcı V. (2003). Bone generation on PHBV matrices: An in vitro study. Biomaterials.

[11] Delaine-Smith R.M., Hann A.J., Green N.H., Reilly G.C. (2021). Electrospun fiber alignment guides osteogenesis and matrix organization differentially in two different osteogenic cell types. Front. Bioeng. Biotechnol..

[12] Zhong L., Hu D., Qu Y., Peng J., Huang K., Lei M., Wu T., Xiao Y., Gu Y., Qian Z. (2019). Preparation of adenosine-loaded electrospun nanofibers and their application in bone regeneration. J. Biomed. Nanotechnol..

[13] Casarin S.A., Malmonge S.M., Kobayashi M., Agnelli J.A.M. (2011). Study on in vitro degradation of bioabsorbable polymers poly(hydroxybutyrate-co-valerate)—(PHBV) and poly(caprolactone)—(PCL). J. Biomater. Nanobiotech..

[14] Kirmanidou Y., Chatzinikolaidou M., Michalakis K., Tsouknidas A. (2024). Clinical translation of polycaprolactone-based tissue engineering scaffolds, fabricated via additive manufacturing: A review of their craniofacial applications. Biomater. Adv..

[15] Gadalla D., Goldstein A.S. (2020). Improving the osteogenicity of PCL fiber substrates by surface-immobilization of bone morphogenic protein-2. Ann. Biomed. Eng..

[16] Baptista-Perianes A., Malmonge S.M., Simbara M.M.O., Santos A.R. (2019). In vitro evaluation of PHBV/PCL blends for bone tissue engineering. Mater. Res..

[17] Rodrigues A.A., Batista N.A., Malmonge S.M., Casarin A.S., Agnelli J.A.M., Santos A.R., Belangero W.D. (2021). Osteogenic differentiation of rat bone mesenchymal stem cells cultured on poly (hydroxybutyrate-co-hydroxyvalerate), poly (ε-caprolactone) scaffolds. J. Mater. Sci. Mater. Med..

[18] (2001). Standard Practice for Direct Contact Cell Culture Evaluation of Materials for Medical Devices.

[19] (2009). Biological Evaluation of Medical Devices. Part 5: Tests for Cytotoxicity: In Vitro Methods.

[20] Al-Maharma A.Y., Patil S.P., Markert B. (2020). Effects of porosity on the mechanical properties of additively manufactured components: A critical review. Mater. Res. Express..

[21] Gregory C.A., Gunn W.G., Peister A., Prockop D.J. (2004). An Alizarin red-based assay of mineralization by adherent cells in culture: Comparison with cetylpyridinium chloride extraction. Anal. Biochem..

[22] Toboga S.R., Vilamaior P.S.L., Carvalho H.F., Recco-Pimentel S.M. (2019). Citoquímica. A Célula.

[23] Vidal B.C., Mello M.L.S. (2019). Toluidine blue staining for cell and tissue biology applications. Acta Histochem..

[24] Modolo L.P., Franca W.R., Simbara M.M.O., Malmonge S.M., Santos A.R. (2023). Dense, porous, and fibrous scaffolds composed of PHBV, PCL, and their 75:25 blend: An morphological and cytochemical characterization. Int. J. Polym. Anal. Charact..

[25] Cheng A., Schwartz Z., Kahn A., Li X., Shao Z., Sun M., Ao Y., Boyan B.D., Chen H. (2019). Advances in porous scaffold design for bone and cartilage tissue engineering and regeneration. Tissue Eng. Part B Rev..

[26] Blokhuis T.J., Arts J.J. (2011). Bioactive and osteoinductive bone graft substitutes: Definitions, facts and myths. Injury.

[27] Oryan A., Alidadi S., Moshiri A., Maffulli N. (2014). Bone regenerative medicine: Classic options, novel strategies, and future directions. J. Orthop. Surg. Res..

[28] Williams J.M., Adewunmi A., Schek R.M., Flanagan C.L., Krebsbach P.H., Feinberg S.E., Hollister S.J., Das S. (2005). Bone tissue engineering using polycaprolactone scaffolds fabricated via selective laser sintering. Biomaterials.

[29] Moroni L., de Wijn J.R., van Blitterswijk C.A. (2006). 3D fiber-deposited scaffolds for tissue engineering: Influence of pores geometry and architecture on dynamic mechanical properties. Biomaterials.

[30] Tubio C.R., Valle X., Carvalho E., Moreira J., Costa P., Correia D.M., Lanceros-Mendez S. (2023). Poly(3-hydroxybutyrate-co-3-hydroxyvalerate) blends with poly (caprolactone) and poly (lactic acid): A comparative study. Polymers.

[31] Prokudina E.S., Senokosova E.A., Antonova L.V., Krivkina E.O., Velikanova E.A., Akentieva T.N., Glushkova T.V., Matveeva V.G., Kochergin N.A. (2023). New tissue-engineered vascular matrix based on regenerated silk fibroin: In vitro study. Sovrem. Tekhnologii Med..

[32] Dalgic A.D., Atila D., Karatas A., Tezcaner A., Keskin D. (2019). Diatom shell incorporated PHBV/PCL-pullulan co-electrospun scaffold for bone tissue engineering. Mater. Sci. Eng. C Mater. Biol. Appl..

[33] Nascimento V.A., Malmonge S.M., Santos A.R. (2023). Culture of rat mesenchymal stem cells on PHBV-PCL scaffolds: Analysis of conditioned culture medium by FT-Raman spectroscopy. Braz. J. Biol..

[34] Zhang Z., Nam H.K., Crouch S., Hatch N.E. (2021). Tissue nonspecific alkaline phosphatase function in bone and muscle progenitor cells: Control of mitochondrial respiration and ATP production. Int. J. Mol. Sci..

[35] Liu L., Hou S., Xu G., Gao J., Mu J., Gao M., He J., Su X., Yang Z., Liu Y. (2024). Evaluation of osteogenic properties of a novel injectable bone-repair material containing strontium in vitro and in vivo. Front. Bioeng. Biotechnol..

[36] San-Marina S., Sharma A., Voss S.G., Janus J.R., Hamilton G.S. (2017). Assessment of scaffolding properties for chondrogenic differentiation of adipose-derived mesenchymal stem cells in nasal reconstruction. JAMA Facial Plast. Surg..

[37] Dec P., Modrzejewski A., Pawlik A. (2022). Existing and novel biomaterials for bone tissue engineering. Int. J. Mol. Sci..

[38] Munhoz M.A.E.S., Pomini K.T., Plepis A.M.G., Martins V.D.C.A., Machado E.G., de Moraes R., Cunha F.B., Santos A.R., Cardoso G.B.C., Duarte M.A.H. (2020). Elastin-derived scaffolding associated or not with bone morphogenetic protein (BMP) or hydroxyapatite (HA) in the repair process of metaphyseal bone defects. PLoS ONE.

[39] Silva S.K., Plepis A.M.G., Martins V.D.C.A., Horn M.M., Buchaim D.V., Buchaim R.L., Pelegrine A.A., Silva V.R., Kudo M.H.M., Fernandes J.F.R. (2022). Suitability of chitosan scaffolds with carbon nanotubes for bone defects treated with photobiomodulation. Int. J. Mol. Sci..

[40] Bonartsev A.P., Voinova V.V., Volkov A.V., Muraev A.A., Boyko E.M., Venediktov A.A., Didenko N.N., Dolgalev A.A. (2022). Scaffolds based on poly(3-hydroxybutyrate) and its copolymers for bone tissue engineering (review). Sovrem. Tekhnologii Med..

[41] Wu C.A., Pettit A.R., Toulson S., Grøndahl L., Mackie E.J., Cassady A.I. (2009). Responses in vivo to purified poly(3-hydroxybutyrate-co-3-hydroxyvalerate) implanted in a murine tibial defect model. J. Biomed. Mater. Res. Part A.

[42] Park S.A., Lee H.J., Kim K.S., Lee S.J., Lee J.T., Kim S.Y., Chang N.H., Park S.Y. (2018). In vivo evaluation of 3D-printed polycaprolactone scaffold implantation combined with β-TCP powder for alveolar bone augmentation in a beagle defect model. Materials.

[43] Lee J.Y., Son S.J., Son J.S., Kang S.S., Choi S.H. (2016). Bone-healing capacity of PCL/PLGA/Duck beak scaffold in critical bone defects in a rabbit model. Biomed. Res. Int..

[44] Gharibshahian M., Salehi M., Beheshtizadeh N., Kamalabadi-Farahani M., Atashi A., Nourbakhsh M.S., Alizadeh M. (2023). Recent advances on 3D-printed PCL-based composite scaffolds for bone tissue engineering. Front. Bioeng. Biotechnol..

[45] Kalaiselvan E., Maiti S.K., Shivaramu S., Banu S.A., Sharun K., Mohan D., Palakkara S., Bag S., Sahoo M., Ramalingam S. (2024). Bone marrow-derived mesenchymal stem cell-laden nanocomposite scaffolds enhance bone regeneration in rabbit critical-size segmental bone defect model. J. Funct. Biomater..

[46] Szczepańczyk P., Szlachta M., Złocista-Szewczyk N., Chłopek J., Pielichowska K. (2021). Recent Developments in Polyurethane-Based Materials for Bone Tissue Engineering. Polymers.

[47] Almansoori A.A., Kwon O.J., Nam J.H., Seo Y.K., Song H.R., Lee J.H. (2021). Mesenchymal stem cells and platelet-rich plasma-impregnated polycaprolactone-β tricalcium phosphate bio-scaffold enhanced bone regeneration around dental implants. Int. J. Implant. Dent..

[48] Jeong W.-S., Kim Y.-C., Min J.-C., Park H.-J., Lee E.-J., Shim J.-H., Choi J.-W. (2022). Clinical Application of 3D-Printed Patient-Specific Polycaprolactone/Beta Tricalcium Phosphate Scaffold for Complex Zygomatico-Maxillary Defects. Polymers.

[49] Helaehil J.V., Lourenço C.B., Huang B., Helaehil L.V., de Camargo I.X., Chiarotto G.B., Santamaria-Jr M., Bártolo P., Caetano G.F. (2022). In Vivo Investigation of Polymer-Ceramic PCL/HA and PCL/β-TCP 3D Composite Scaffolds and Electrical Stimulation for Bone Regeneration. Polymers.

[50] Bassi A.P.F., Bizelli V.F., Francatti T.M., Rezende de Moares Ferreira A.C., Carvalho Pereira J., Al-Sharani H.M., de Almeida Lucas F., Faverani L.P. (2021). Bone Regeneration Assessment of Polycaprolactone Membrane on Critical-Size Defects in Rat Calvaria. Membranes.

